# County-Level Ecological Association Between Glyphosate Exposure and Chronic Disease Mortality in Midwestern US Agricultural Communities

**DOI:** 10.7759/cureus.106908

**Published:** 2026-04-12

**Authors:** Alex N Egbuchiem, Victor C Ezeamii

**Affiliations:** 1 Public Health Research, University of Nebraska Medical Center (UNMC) College of Public Health, Omaha, USA; 2 Public Health, Georgia Southern University Jiann-Ping Hsu College of Public Health, Statesboro, USA

**Keywords:** agricultural exposure, cdc wonder, chronic disease, glyphosate, heart disease, mortality

## Abstract

Background: Glyphosate is widely used in the United States (US) agriculture, particularly in regions with intensive crop production. Concerns have been raised regarding potential long-term health effects associated with environmental exposure. Chronic diseases remain a leading cause of mortality in the US, particularly in agricultural and rural communities.

Objective: To examine the ecological association between county-level glyphosate use and chronic disease mortality in Midwestern US agricultural communities.

Methods: This ecological panel study used county-level mortality data from the Centers for Disease Control and Prevention (CDC) Wide-ranging Online Data for Epidemiologic Research (WONDER) Multiple Cause of Death files and pesticide use estimates from the United States Geological Survey Pesticide National Synthesis Project. The study included counties across 12 Midwestern states from 1999 to 2020. Age-adjusted mortality rates for cancer, heart disease, diabetes, Alzheimer's disease, kidney disease, and chronic respiratory disease were analyzed. The primary exposure was log-transformed glyphosate use scaled per 1,000 kg and lagged by three years. County and year fixed effects regression models were applied.

Results: Higher three-year lagged glyphosate use was significantly associated with increased heart disease mortality at the county level (β = 5.04; p < 0.001), indicating that increases in glyphosate exposure were associated with modest increases in county-level heart disease mortality rates. No significant associations were observed for the other chronic disease outcomes.

Conclusion: This study highlights a population-level association between glyphosate use and heart disease mortality in Midwestern counties. Continued monitoring and research using individual-level data are warranted.

## Introduction

Over the past 20 years, there has been a great transformation in the timing and method of applying the glyphosate herbicides and a tremendous increase in the overall amount applied in the United States (US) [1]. Glyphosate, which is in the market since the mid-1970s, is still being used as one of the most commonly used herbicides worldwide [2]. Plantations and herbicide-tolerant (HT) genetically modified (HT GM) producers have been increasingly encouraged to add other herbicides (with other and complementary modes of action) to glyphosate and, in some instances, to revert to ploughing in more integrated weed control regimes [3]. This has contributed to the fact that the Midwestern US, also referred to as the Corn Belt, has recorded some of the worst cases of glyphosate application in the world [4]. Even though glyphosate has helped farmers record high productivity and efficiency within their farms, glyphosate-based herbicides (GBHs), in most cases, contaminate the drinking water sources, precipitation, and air, particularly in the agricultural areas [5]. Glyphosate exposure has been associated with respiratory symptoms in some studies, raising concerns about potential chronic respiratory effects in agricultural settings.

Glyphosate is widely present in the environment, and human exposure occurs through multiple pathways, including dietary intake from both plant-based and non-plant-based products [6]. Glyphosate exposure has been associated with respiratory symptoms in some studies, although evidence regarding long-term respiratory effects remains limited [7]. Although glyphosate is generally considered to have relatively low acute toxicity compared to other herbicides, there is growing concern regarding the potential long-term health effects of repeated exposure [8,9].

Chronic diseases are the most common causes of death in the US, and they constitute a significant percentage of deaths and health expenditure [10,11]. Most of the untimely deaths in the country are caused by conditions such as cardiovascular disease, cancer, chronic respiratory diseases, and metabolic disorders [12]. In rural and agricultural areas, the environment, occupation, socioeconomic status, and healthcare access factors may interact in a complicated way to affect patterns of chronic disease mortality [13]. Pesticides, fertilizers, and other agrochemicals have a tendency to expose agricultural workers and the rural population to unique exposures, and this could be one of the reasons why the health outcomes may vary according to geographic locations [14,15].

Even though there has been increasing curiosity in the possible health impact of glyphosate, much of the current research has been on the occupational exposure of individuals at work, or toxicological evaluations of glyphosate at test-tube levels [16]. Even less research has studied the larger population-scale patterns that establish correlations between the intensity of environmental exposure and health outcomes at the geographic areas [17,18].

The study will utilize publicly available datasets from the United States Geological Survey (USGS) and the Centers for Disease Control and Prevention (CDC). The USGS data keep very specific estimates of the county-wide pesticide application, such as the glyphosate application in the main crop-producing areas [19]. CDC mortality databases are primary sources of standardized information on cause-specific county-wide deaths in the US [20]. Combining these datasets allows for formulating analytical frameworks that can be used to test the relationships between patterns of environmental exposure and changes in population health results.

The objective of this study was to assess the ecological association between county-level glyphosate application intensity and chronic disease mortality across Midwestern US agricultural communities using USGS and CDC data sources. The findings of the study will make a contribution to the understanding of the environmental determinants of chronic diseases.

## Materials and methods

Study design and data source

This study employed a longitudinal ecological panel design to examine county-level associations between agricultural glyphosate exposure and chronic disease mortality in Midwestern US agricultural communities. Annual county-level mortality data for the period 1999 through 2020 were obtained from the CDC Wide-ranging Online Data for Epidemiologic Research (CDC WONDER) Multiple Cause of Death Database [21].

Mortality data were extracted from the Multiple Cause of Death files using the underlying cause of death (UCD) field. Queries were restricted to the 12 Midwestern states of interest and grouped by county of residence and year of death. All ages, both sexes, and all race categories were included. Age-adjusted mortality rates per 100,000 population were calculated using the 2000 United States Standard Population. Results were exported with zero values displayed and suppressed values excluded. County-level data were then downloaded for each disease category separately and merged with exposure data by county code and year. County-level agricultural glyphosate use estimates were obtained from the USGS Pesticide National Synthesis Project [22]. All datasets are publicly available. Data were merged by five-digit Federal Information Processing Standard (FIPS) county codes and calendar year. The final analytic dataset used for regression analyses with three-year lagged exposure contained 17,823 county-year observations.

Study population

The study population consisted of counties located in 12 Midwestern states: Illinois (17), Indiana (18), Iowa (19), Kansas (20), Michigan (26), Minnesota (27), Missouri (29), Nebraska (31), North Dakota (38), Ohio (39), South Dakota (46), and Wisconsin (55). Each county-year constituted one observation. Counties were included if both mortality data and glyphosate exposure estimates were available for the corresponding year and if exposure could be lagged by three years. The unit of analysis was the county-year. Individual-level data were not used.

Variables and measures

The primary exposure was county-level annual glyphosate use measured in kilograms. Exposure values were scaled per 1,000 kilograms prior to log transformation to improve interpretability and address right skewness. A three-year lag exposure variable was constructed within each county to reflect potential latency between agricultural exposure and mortality outcomes. The main independent variable in all regression models was the log-transformed three-year lagged glyphosate exposure.

Outcome variables were county-level age-adjusted mortality rates per 100,000 population for six chronic disease categories. Cancer mortality was defined as deaths where the UCD was coded in the International Classification of Diseases, 10th Revision (ICD‑10) as malignant neoplasms (codes C00-C97), according to the standard 113‑cause list. Heart disease mortality was defined using codes I00-I09, I11, I13, and I20-I51. Diabetes mortality was defined using codes E10-E14. Alzheimer's disease mortality was defined using code G30. Kidney disease mortality was defined using codes N00-N07, N17-N19, and N25-N27. Chronic lower respiratory disease mortality was defined using codes J40-J47. Cause of death groupings followed the selected UCD categories provided by the data source.

Missing data

Mortality rates flagged by the data source as unreliable or suppressed due to small counts were coded as missing. Missingness occurred primarily in counties with small populations or low event numbers. Glyphosate exposure data were complete for the available years. Observations with missing outcome or exposure values were excluded from regression analyses using outcome-specific complete case analysis. The final analytic dataset for the three-year lag exposure models included 17,823 county-year observations, with sample sizes varying across disease-specific models due to differential outcome missingness. No imputation procedures were performed.

Statistical analysis

Descriptive statistics were calculated for exposure and outcome variables using available county-year observations. Associations between glyphosate exposure and age-adjusted mortality rates were estimated using two-way fixed effects linear regression models. Each model included county fixed effects to control for time-invariant county characteristics and year fixed effects to account for secular temporal trends. The primary exposure variable was the log-transformed three-year lagged glyphosate use scaled per 1,000 kg. Log transformation was applied to reduce right skewness in the exposure distribution, stabilize variance, and improve model fit by approximating linearity between glyphosate use and mortality rates. No additional time-varying covariates were included in the models. This approach was selected due to limitations in the consistent availability of county-level socioeconomic and behavioral data across the full study period and to avoid over-adjustment within the ecological framework. Standard errors were clustered at the county level to account for within-county correlation over time. Separate regression models were estimated for each mortality outcome. Statistical significance was evaluated using two-sided tests with an alpha level of 0.05. All analyses were conducted using R (version 4.5; R Development Core Team, Vienna, Austria).

Ethical considerations

This study used publicly available, deidentified, aggregate county-level data. No individual-level information was accessed, and no human subjects were directly involved. Institutional review board approval was not required because the study analyzed secondary publicly available data without personal identifiers.

## Results

Table 1 presents the descriptive characteristics of county-level mortality rates and glyphosate exposure across Midwestern counties during the study period.

**Table 1:** Descriptive statistics of county-level variables (1999-2020) Mortality rates are age-adjusted per 100,000 population using the 2000 US Standard Population and derived from CDC WONDER Multiple Cause of Death data. Glyphosate exposure represents annual county-level agricultural use in kilograms derived from the United States Geological Survey Pesticide National Synthesis Project. The log-transformed exposure reflects a three-year lag and was scaled per 1,000 kg prior to transformation. Values are reported as mean and standard deviation. SD denotes standard deviation.

Variable	Mean (SD)
Cancer Mortality Rate (per 100,000)	180.60 (32.23)
Heart Disease Mortality Rate (per 100,000)	195.84 (50.84)
Diabetes Mortality Rate (per 100,000)	27.39 (10.71)
Alzheimer’s Mortality Rate (per 100,000)	34.78 (12.84)
Kidney Disease Mortality Rate (per 100,000)	17.67 (7.10)
Chronic Respiratory Mortality Rate (per 100,000)	53.78 (16.14)
Glyphosate Use (kg per county-year)	59,721.11 (57,583.33)
Log-Transformed Glyphosate (3-year lag)	3.43 (1.32)

The results indicate that heart disease had the highest mean mortality rate at 195.84 (50.84) per 100,000, followed by cancer at 180.60 (32.23). Chronic respiratory disease mortality averaged 53.78 (16.14), while Alzheimer's disease averaged 34.78 (12.84). Diabetes and kidney disease showed lower mean mortality rates at 27.39 (10.71) and 17.67 (7.10), respectively. Mean annual glyphosate use was 59,721.11 (57,583.33) kg per county-year, indicating substantial variability across counties and years. The mean of the log-transformed three-year lag exposure was 3.43 (1.32), reflecting the distribution after scaling and transformation.

Table 2 presents the fixed effects regression estimates examining the association between three-year lagged glyphosate exposure and county-level age-adjusted mortality rates.

**Table 2:** Association between log-transformed three-year lagged glyphosate use and county-level age-adjusted mortality rates Outcome variables are county-level age-adjusted mortality rates per 100,000 population for cancer, heart disease, diabetes, Alzheimer's disease, kidney disease, and chronic lower respiratory disease, derived from CDC WONDER and standardized to the 2000 United States Standard Population. Exposure represents log-transformed glyphosate use scaled per 1,000 kg and lagged by three years. All models include county and year fixed effects. Standard errors are shown in parentheses and are clustered at the county level. The number of observations differs across outcomes because some county-year mortality rates were suppressed or classified as unreliable by CDC WONDER and were excluded from analysis. Within R squared reflects the proportion of within county variation explained by the model. Statistical significance is indicated by asterisks, where *p < 0.05, **p < 0.01, and ***p < 0.001.

Variable	Cancer	Heart Disease	Diabetes	Alzheimer's	Kidney Disease	Respiratory Disease
Log Glyphosate (3-Year Lag)	1.179 (0.797)	5.035*** (1.110)	-0.013 (0.489)	-1.059 (0.647)	0.563 (0.429)	-0.962 (0.555)
Observations	15,298	15,982	3466	4569	2210	7378
Within R²	0.000	0.002	0.000	0.001	0.002	0.001
County FE	Yes	Yes	Yes	Yes	Yes	Yes
Year FE	Yes	Yes	Yes	Yes	Yes	Yes

The results indicate that higher three-year lagged glyphosate exposure was statistically associated with heart disease mortality (β = 5.04; p < 0.001). No statistically significant associations were observed for cancer, diabetes, Alzheimer's disease, kidney disease, or respiratory disease at the 0.05 level. The magnitude of the estimated coefficients varied across outcomes, but most were small and imprecisely estimated. Given that multiple outcomes were examined, the possibility of chance findings due to multiple comparisons should be considered when interpreting the statistically significant association with heart disease. Within R² values were low across models, indicating limited variation explained by changes in exposure within counties over time, which is expected in ecological analyses of complex, multifactorial health outcomes influenced by numerous unmeasured factors. Sample sizes ranged from 2,210 for kidney disease to 15,982 for heart disease, reflecting differences in outcome availability.

**Figure 1:**
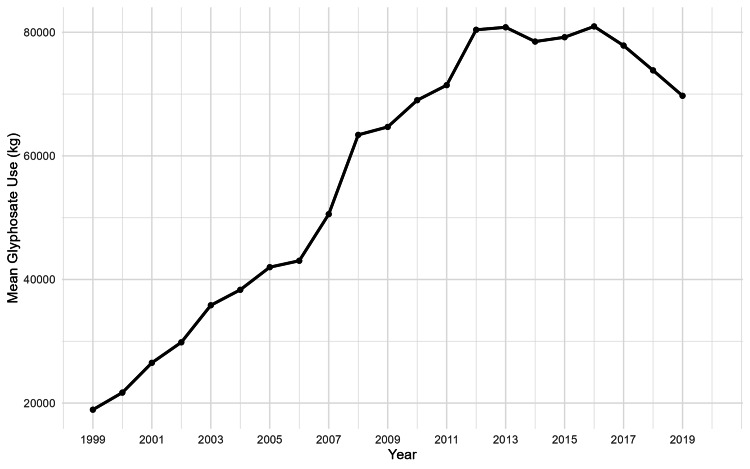
Mean annual county-level glyphosate use in kilograms (1999-2020) Values represent the average annual glyphosate use in kilograms per county across the 12 Midwestern states included in the study. Data were obtained from the United States Geological Survey Pesticide National Synthesis Project. The final year reflects available exposure data for that calendar year.

Figure 1 illustrates the temporal trend in mean annual county-level glyphosate use from 1999 to 2020.

The figure shows a steady increase in glyphosate application between 1999 and approximately 2012. Mean use rose from below 20,000 kg per county-year in 1999 to more than 80,000 kg by 2012. Between 2012 and 2016, glyphosate use remained relatively stable at high levels, fluctuating around 78,000-82,000 kg. After 2016, a gradual decline is observed through 2019, with mean use decreasing to approximately 70,000 kg.

The pattern indicates sustained growth in glyphosate use during the early and middle years of the study period, followed by stabilization and a modest reduction in later years.

## Discussion

This study examined county-level glyphosate use and age-adjusted mortality from selected chronic diseases in Midwestern agricultural communities. The primary finding was a statistically significant association between three-year lagged glyphosate exposure and heart disease mortality, while no statistically significant associations were observed for cancer, diabetes, Alzheimer's disease, kidney disease, or chronic respiratory disease. Glyphosate use increased substantially from 1999 through the early 2010s, followed by stabilization and modest decline. These findings are relevant in light of documented expansion in glyphosate application in the US during the widespread adoption of glyphosate-resistant crops [1,4]. Rising agricultural use has been linked to changing pesticide practices and crop management patterns [3,4]. Human biomonitoring studies have confirmed measurable glyphosate exposure in agricultural workers and in the general population [2,7,17]. Reviews have described potential human health concerns related to glyphosate-based herbicides, including possible effects on cardiovascular, neurologic, and metabolic systems [5,6,8]. The observed association with heart disease mortality is consistent with evidence that chronic diseases remain a major public health burden in the US [10-12]. Cardiovascular mortality trends have been extensively monitored using CDC WONDER data, which provide reliable age-adjusted rates for population-level assessment [20,21]. While this study does not establish causation, the findings align with ongoing concerns about environmental exposures and chronic disease patterns in agricultural settings [14,15,18].

Current US public health guidance emphasizes the reduction of preventable chronic disease risk factors and monitoring of environmental exposures. National efforts to eliminate leading causes of premature death include attention to cardiovascular disease prevention and environmental health protection [12]. Surveillance systems, such as CDC WONDER, support population-level monitoring of mortality trends and disparities [21]. The burden of chronic disease is particularly important in rural areas, where aging populations and healthcare access challenges may influence outcomes [13]. Federal pesticide use monitoring through the USGS provides transparency regarding agricultural chemical application and supports environmental health assessment [22]. These frameworks emphasize the importance of examining environmental exposures within broader chronic disease prevention strategies.

Several biological pathways have been discussed in the literature that may relate to glyphosate exposure and chronic disease outcomes. Experimental and observational studies have described oxidative stress responses associated with glyphosate exposure [16]. Reviews have also reported possible effects on endocrine signaling, immune regulation, and neural function [6,8,9]. Oxidative stress and inflammation are recognized contributors to cardiovascular disease development [10,11]. Biomonitoring data have demonstrated measurable urinary glyphosate concentrations in agricultural populations, suggesting systemic absorption following environmental exposure [2,7,17]. Environmental assessments indicate that residents living near agricultural land may experience indirect exposure through air, water, and soil pathways [14,18]. These mechanisms provide a reasonable context for the observed association with heart disease mortality, although ecological data cannot determine individual-level biological effects.

Strengths and limitations of the study

This study has several strengths and limitations. Strengths include the use of standardized age-adjusted mortality data from CDC WONDER and county-level pesticide use estimates from the USGS, allowing consistent assessment of exposure and outcomes across multiple states over an extended period. The longitudinal panel structure and two-way fixed effects approach help account for time-invariant county characteristics and shared temporal trends, improving internal consistency at the population level.

Several limitations should be considered. The analysis relies on aggregated county-level exposure estimates rather than individual-level measurements, and the ecological design limits interpretation at the individual level. Some mortality observations were excluded because CDC WONDER flagged them as unreliable, resulting in variation in sample size across outcomes. Pesticide use estimates are based on agricultural reporting and may be subject to measurement error. In addition, the structure of the data across repeated years does not allow clear temporal sequencing beyond the applied lag.

Within R² values were low across models, indicating that only a small proportion of within-county variation in mortality rates is explained by changes in glyphosate exposure over time. This suggests that most of the variation is likely driven by other unmeasured or multifactorial influences, including demographic shifts, healthcare access, and behavioral risk factors. As such, the observed associations should be interpreted cautiously and not viewed as strong explanatory effects at the population level. Residual confounding remains an important consideration, particularly because the models did not include detailed time-varying covariates, such as changes in healthcare access, lifestyle behaviors, environmental exposures, or local economic conditions over time. These factors may vary within counties across years and could influence both glyphosate use patterns and mortality outcomes, potentially affecting the observed associations. The models did not adjust for multiple comparisons across the outcomes examined, and results should be interpreted with this in mind. It is also possible that neighboring counties share similar environmental, agricultural, and demographic characteristics; this kind of spatial correlation was not explicitly modeled and may affect the independence of observations.

Future work should incorporate individual-level exposure data, additional time-varying covariates, and approaches that better account for geographic patterns, along with study designs that allow a clearer understanding of temporal relationships and population differences.

## Conclusions

This study demonstrates an ecological association between county-level glyphosate use and heart disease mortality in Midwestern US agricultural communities, while no significant associations were observed for cancer, diabetes, Alzheimer's disease, kidney disease, or respiratory disease. These findings are associative and should be interpreted as hypothesis-generating rather than causal. The results provide population-level context for ongoing discussions on environmental exposures and chronic disease patterns in the US. Given the widespread use of glyphosate and the high burden of cardiovascular disease, continued monitoring of agricultural chemical use and health outcomes remains important. Future studies incorporating individual-level exposure assessment, detailed socioeconomic and behavioral factors, and longitudinal designs are needed to better evaluate temporal relationships and potential underlying mechanisms.
